# Supplementary material for: Precise control of embolic stroke with magnetized red blood cells in mice
Source: Commun Biol. 2022 Feb 16;5:136. doi: 10.1038/s42003-022-03082-9 (PMC8850623; doi:10.1038/s42003-022-03082-9)
Supplement: Supplementary file 2 — Supplementary information [file 42003_2022_3082_MOESM2_ESM.pdf]

Supplementary Information

Precise control of embolic stroke with magnetized red blood cells in mice

Yuxiao Jin[1,2,3,4], Peijun Shi[5,6], Yu Wang[7], Jinghang Li[6,8], Jiachen Zhang[9], Xinxin Zhao[10], Yaping Ge[2,3,4], Yanjie Huang[2], Mengzhun Guo[7,11], Feidi Wang[12], Bo Ci[13], Xian Xiao[2,3], Xiaofei Gao[2,3], Jianrong Xu[10], Bobo Dang[7,11], Botao Ji[5,6], Woo-ping Ge[14] and Jie-Min Jia[2,3,4]* (* corresponding author)

[1]College of Life Sciences, Zhejiang University, Hangzhou, Zhejiang, 310058, China.

[2]Key Laboratory of Growth Regulation and Translational Research of Zhejiang Province, School of Life Sciences, Westlake University, Hangzhou, 310024, China.

[3]Westlake Laboratory of Life Sciences and Biomedicine, Hangzhou, 310024, China

[4]Laboratory of Neurovascular Biology, Institute of Basic Medical Sciences, Westlake Institute for Advanced Study, Hangzhou, 310024, China.

[5]Key Laboratory of 3D Micro/Nano Fabrication and Characterization of Zhejiang Province, Hangzhou, China.

[6]School of Engineering, Westlake University and Institute of Advanced Technology, Westlake Institute for Advanced Study, Hangzhou, China.

[7]Zhejiang Provincial Laboratory of Life Sciences and Biomedicine, Key Laboratory of Structural Biology of Zhejiang Province, Westlake University, Hangzhou, China.

[8]School of Materials Science and Engineering, Wuhan Institute of Technology, Wuhan, China.

[9]School of Basic Medical Sciences, Wuhan University, Wuhan, China.

[10]Department of Radiology, Ren Ji Hospital, School of Medicine, Shanghai Jiao Tong University, Shanghai, China.

[11]Institute of Biology, Westlake Institute for Advanced Study, Hangzhou, China.

[12]Departments of Anesthesiology & Center for Brain Science, The First Affiliated Hospital of Xi'an Jiao Tong University, Xi'an, Shaanxi Province, China.

[13]Children's Medical Center Research Institute, University of Texas Southwestern Medical Center, Dallas, TX, USA

[14]Chinese Institute for Brain Research, Beijing, China

*Correspondence: jiajiemin@westlake.edu.cn

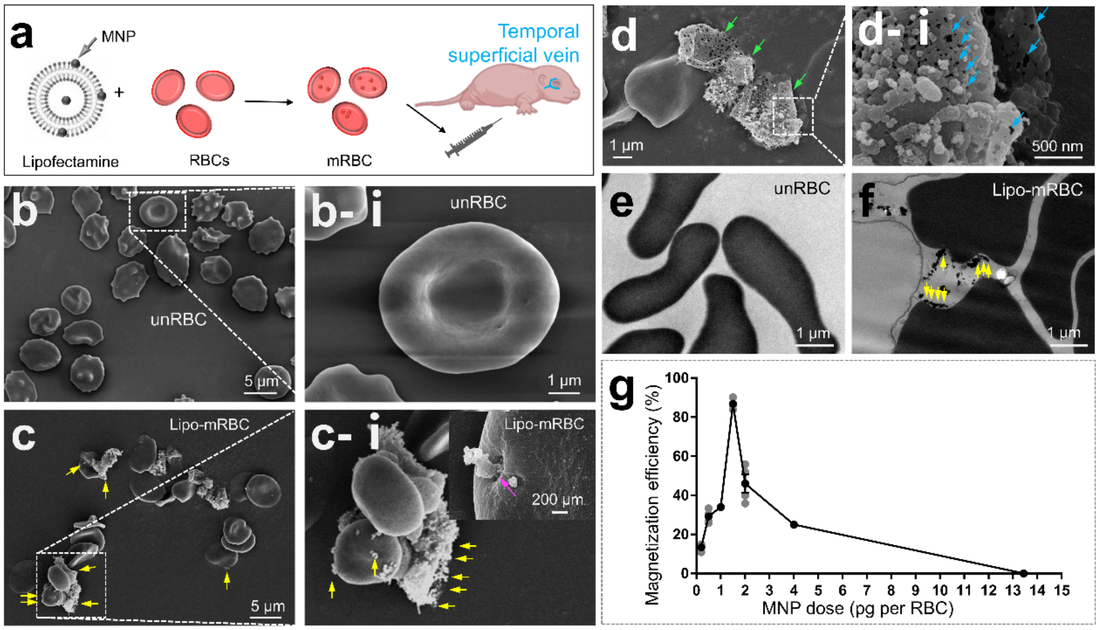

Supplementary Figure 1: Lipofectamine-mediated mRBC (Lipo-mRBC) production and magnetization efficiency dose curve. MNPs with an average size of 180 nm (MicroMod, Germany) were used in this paradigm. (**a**) Schematic illustration of the binding between MNPs and RBCs through Lipofectamine. (**b**-**f**) SEM (**b**-**b- i** , **c**-**c- i** , **d**-**d- i**) and TEM (**e**-**f**) were conducted to evaluate the binding of MNPs to RBCs. Typical images show unmagnetized RBCs (unRBC) in the control group (**b**-**b- i** , **e**) or the binding of MNPs to magnetized RBCs at low (**c**-**c- i** , **f**) and high (**d**-**d- i**) MNP doses. Yellow arrows indicate MNPs that bound to or embedded into the membrane of RBCs. Magenta arrow in **c- i** shows the RBC membrane dint. Green arrows in **d** point to damaged RBCs, and blue arrows point to holes in the RBC membrane. (**g**) The magnetization efficiency of RBCs at different doses of MNPs, N = 3 for 0.2 pg per RBC, N = 4 for 0.5 and 2 pg per RBC, N = 3 for 1.5 pg per RBC. There were two data points (0.5 and 1.5 pg per RBC) shared with Fig. 1**h** in this dataset, and N = 1 for the remaining conditions. N indicates biological replicates. Data are presented as mean ± SEM.

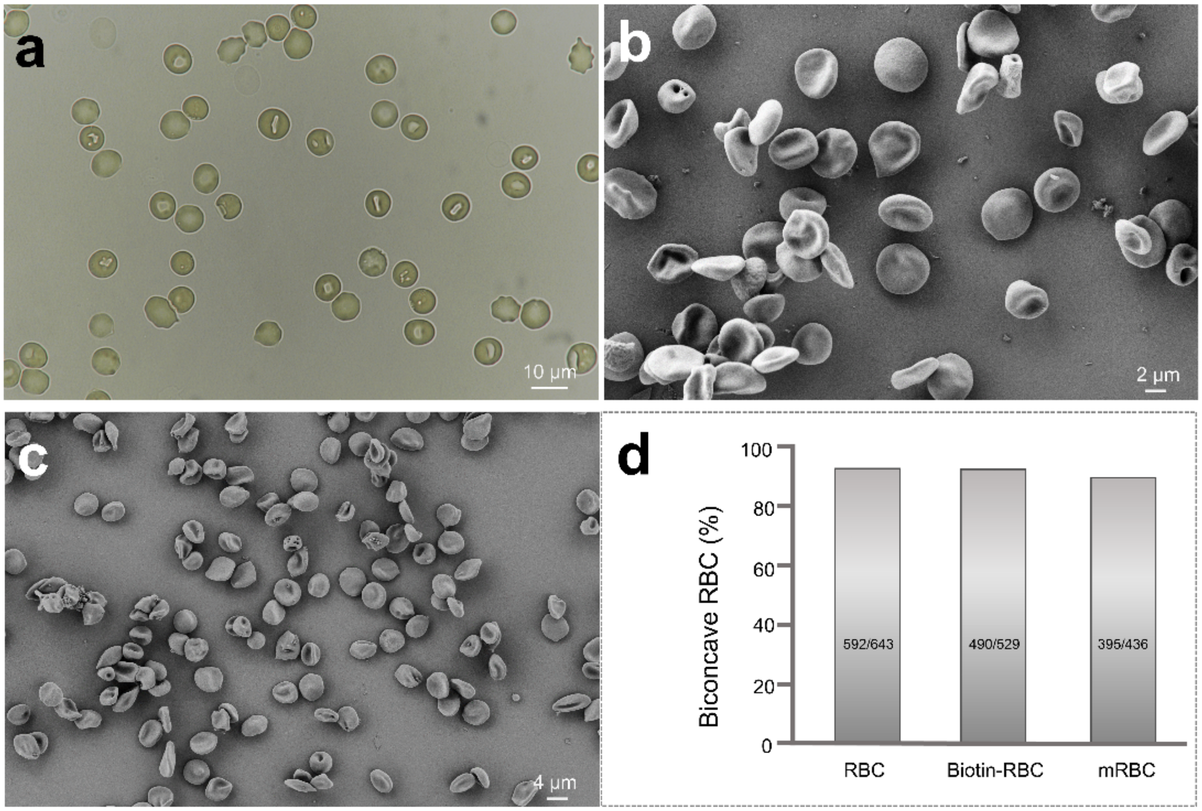

Supplementary Figure 2: RBC quality monitoring during mRBC preparation. (**a**) A representative bright-field micrograph of the washed RBCs prior to the antibody incubation step; 592 out of 643 RBCs were biconcave in shape. (**b**) Scanning electron micrograph of RBCs coated with an antibody that specifically binds to RBCs (biotinylated anti-Ter119 antibody); 490 out of 529 RBCs were biconcave in shape. (**c**) Scanning electron micrograph of the final magnetized RBCs; 395 out of 436 RBCs were biconcave in shape. (**d**). Quantification of the percentages of RBCs with a biconcave shape in each step, n = 643 cells, 529 cells, 436 cells for each group.

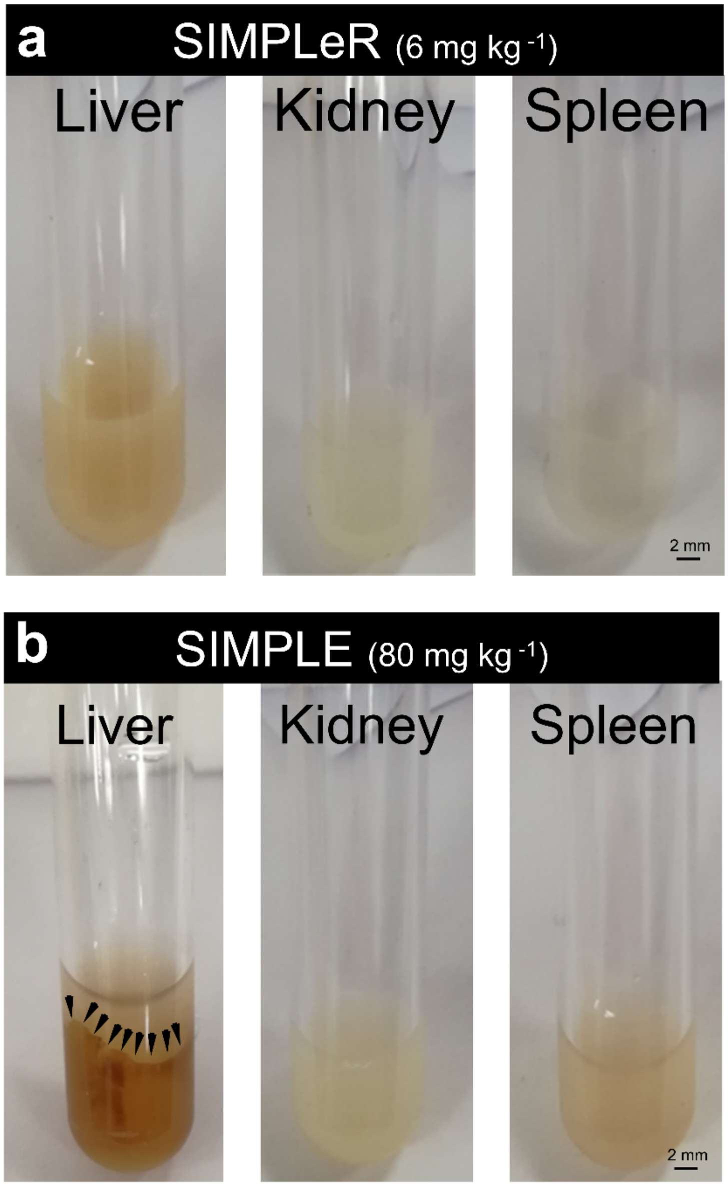

Supplementary Figure 3: Homogenates from mouse pup liver, kidney and spleen. Mouse pups either underwent SIMPLeR (6 mg kg^{-1}) in **a** or SIMPLE (80 mg kg^{-1}) in **b**. Visible MNPs (indicated by the black arrow in **b**) were separated by using a 1-well magnet frame (EasySep™ Magnet). However, MNP in **a** was not detectable. The total protein amounts were comparable in the corresponding organs between **a** and **b**. The experiment was repeated for 4 times by using with 4 mice.

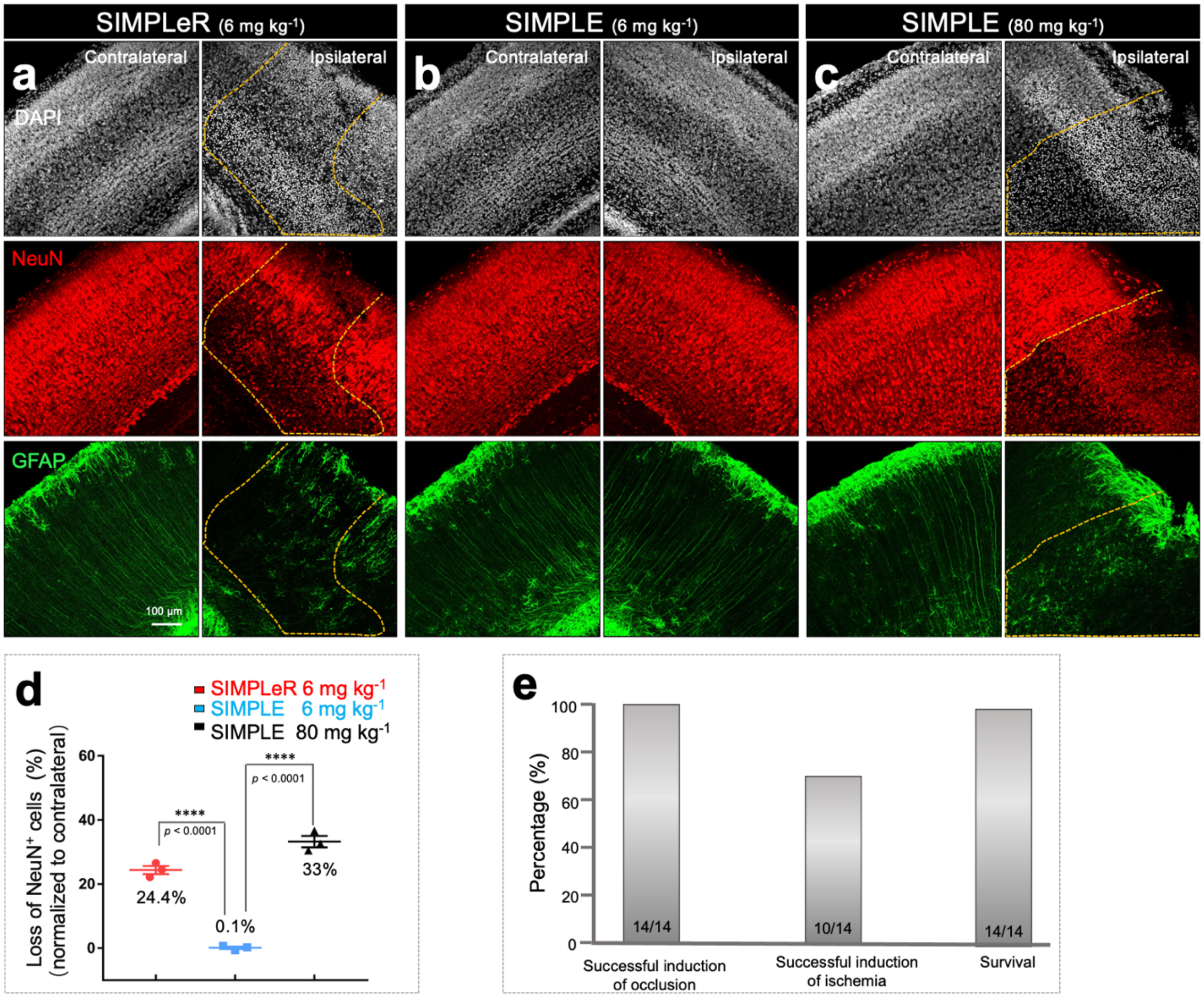

Supplementary Figure 4: SIMPLeR induces nerve cell loss in the somatosensory cortex. (**a-c**) Confocal images of ipsilateral and contralateral P3 mouse somatosensory cortex. These pups were insulted by 6-hour SIMPLeR (**a**) and SIMPLE (**b-c**). Hoechst counterstaining and immunostaining of NeuN and GFAP revealed shrunken nuclei, lost neurons, and damaged radial glia processes. The yellow dotted line shows the core area of stroke. (**d**) The proportion of $NeuN^+$ cell loss in **a**, normalized to the contralateral side, N = 3 mice for each condition. The effect sizes of SIMPLeR and SIMPLE (80 mg kg^{-1}) relative to the reference group SIMPLE (6 mg kg^{-1}) are 44 and 60 respectively. (**e**) The rate of successful occlusion formation, cerebral ischemia induction, and survival rate of mouse pups that were subjected to SIMPLeR. N = 14 mouse pups for each group. Data are presented as mean ± SEM.

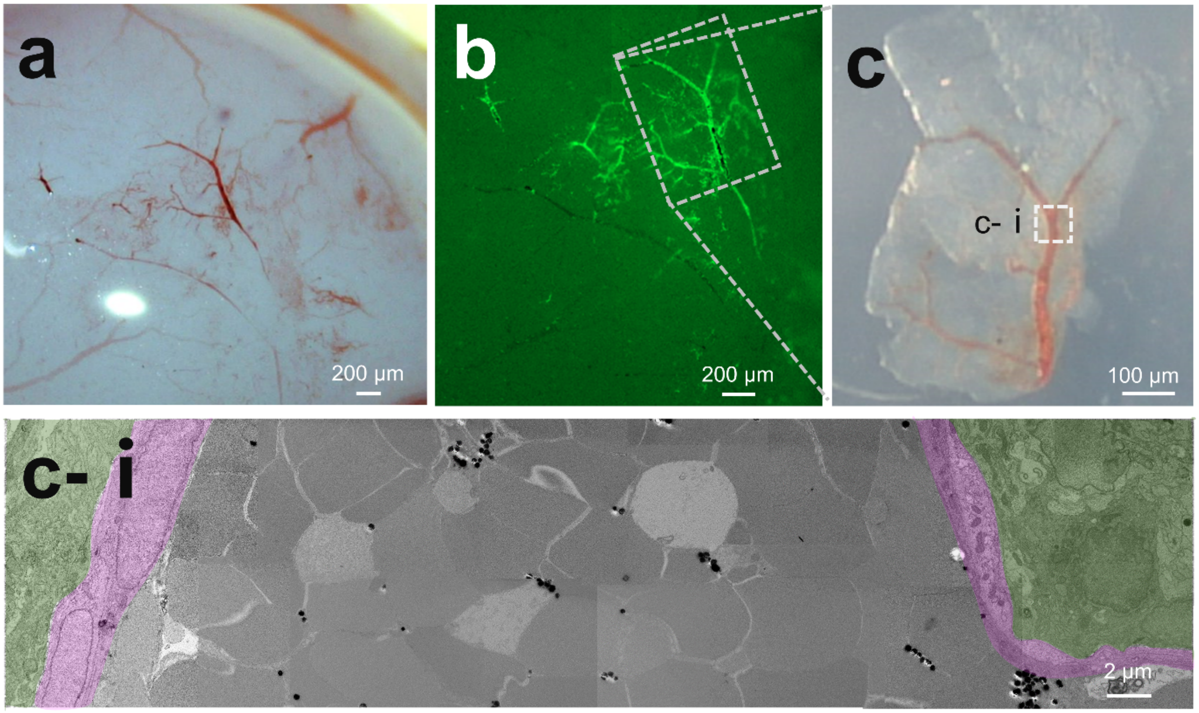

Supplementary Figure 5: TEM sample preparation for SIMPLeR-induced emboli. (**a**-**c**) Bright field (**a**) and fluorescent stereoscopic images (**b**) of a P3 mouse brain with dMCA occluded by Alexa Fluor 488-mRBCs, which is shown in Fig. 2**g**. (**c- i**) The segment of dMCA in the white box in **c** was used for EM sample preparation.

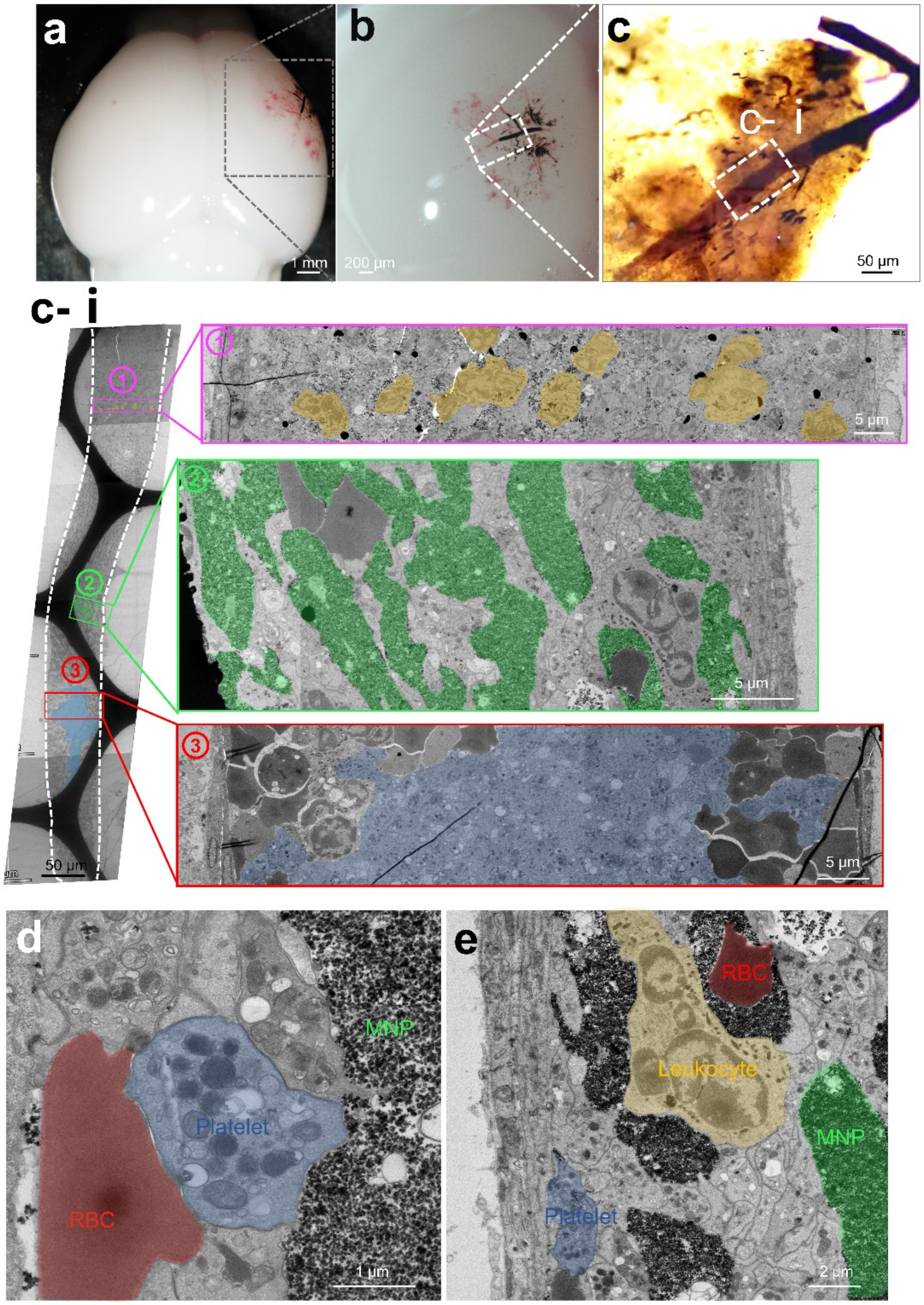

Supplementary Figure 6: TEM for white-thrombus-like structures induced by SIMPLE in P3 mouse pups. (**a**-**c**) SIMPLE mediated dMCA occlusion for 6 hours. The occluded segment of the dMCA (**b**) was dissected for EM sample preparation (**c**). (**c**- ⅰ) Picture of the dMCA segment deposited on the copper grid. (①-③) High-magnification TEM images of the MNP-rich region (②), white blood cell–rich region (①) and

platelet-rich region (③). (**d**-**e**) High magnification images showing the RBCs, platelets, MNPs and leukocytes in ②. Leukocytes are highlighted in yellow; MNPs are highlighted in green; platelets are highlighted in blue; RBCs are highlighted in red.

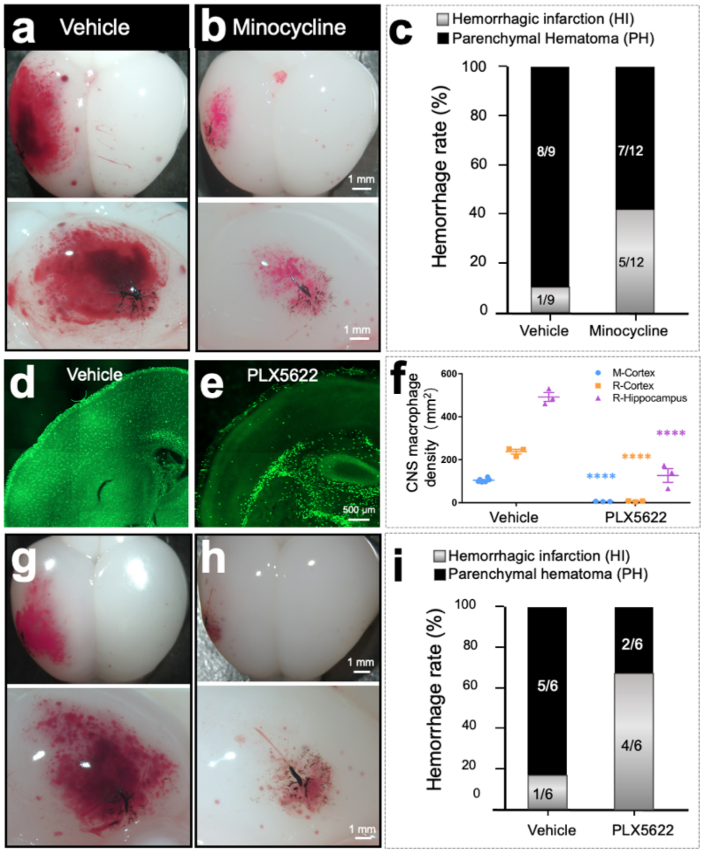

Supplementary Figure 7: Inhibition or elimination of macrophages in rats reduced the degree of bleeding. (**a**-**b**) Representative bright-field images of brains of P2-4 rat pups that were intraperitoneally injected and treated with vehicle (**a**) or minocycline **b**) prior to 6-hour SIMPLE insult. (**c**) Quantification of the percentages of mild HI-type and more severe PH-type hemorrhagic transformation for **a** and **b**. N = 9 rats for vehicle group and 12 rats for minocycline treated group. (**d**-**e**) Representative confocal images of brain sections from rat brains in **g** and **h**. The central nervous system (CNS) macrophage marker Iba1 is shown as a green fluorescence signal. (**f**) The CNS macrophage density was quantitatively analyzed for **d**-**e** in rats and Figure 4**i** in mice. N = 3 for each group. The effect sizes of differences in macrophage density in PLX 5622 treatment relative to the vehicle treated group in mouse cortex (M-cortex), rat cortex (R-cortex) and rat hippocampus (R-Hippocampus) are 12.3, 14.5, and 12.7 respectively. (**g**-**h**) Bright-field pictures of brains from rats that were treated with either vehicle or PLX 5622 prior to the 6-hour occlusion insult. (**i**) Quantification of the degree of hemorrhagic transformation in rats for **g** and **h,** N = 6 rats for each condition. Gray indicates the mild HI type, while black indicates the more severe PH type. The difference in macrophage density was compared between vehicle and PLX5622 treated groups for the same brain region. ****, $p < 0.0001$. Data are presented as mean SEM.
