# Supplementary material for: Precise control of embolic stroke with magnetized red blood cells in mice
Source: Commun Biol. 2022 Feb 16;5:136. doi: 10.1038/s42003-022-03082-9 (PMC8850623; doi:10.1038/s42003-022-03082-9)
Supplement: Supplementary file 3 — Description of Additional Supplementary Files [file 42003_2022_3082_MOESM3_ESM.pdf]

Description of Additional Supplementary Files

File name: Supplementary Movies

Description:

Movie 1. MNPs aggregated into a line on the edge a magnet that is outlined with a dashed box.

Movie 2. mRBCs aggregated into a line on the edge a magnet that is outlined with a dashed box.

Movie 3. Unmagnetized RBCs remain in a steady state when placed on top of a magnet.

Movie 4. Magnet trapped DiO-mRBCs in vitro.

Movie 5. SIMPLeR fully occlude the distal middle cerebral artery in P3 mouse brain. DiO-mRBCs aggregated at the targeted vessel (magenta arrows), while green DiO-RBCs cannot enter this occluded branch (magenta dashed line); instead, they took a detour through another branch from the parental vessels (red dashed line). Twenty-three million of mRBCs were injected.

Movie 6. mRBC aggregation was reversible following the removal of the magnet. The moving blue arrowheads trace the track of mRBCs when they were undergone dispersion.

Movie 7. SIMPLE disrupted the blood flow direction in the distal middle cerebral artery of CX3CR1-EGFP mouse pups at P6, which was recorded by two-photon microscopy. Central nervous system macrophages are labeled with green fluorescent protein in this engineered mouse line. Red arrow indicates the blood flow directions.

Movie 8. Liver pieces of P4 mouse pup that subjected to a 6-hour SIMPLeR did not move accordingly when the magnet was moving (red arrow).

Movie 9. Spleen pieces of P4 mouse pup that subjected to a 6-hour SIMPLeR did not move accordingly when the magnet was moving (red arrow).

Movie 10. Liver pieces of mouse pup were moving according to the changing magnetic gradient (red arrow). This P4 mouse pup was subjected to SIMPLE (80 mg kg-1), and its liver was dissected, cut into pieces, and placed into a 24-well plate. The moving magnet led to a changing magnetic gradient.

Movie 11. Spleen pieces of mouse pup were moving according to the changing magnetic gradient (red arrow). This P4 mouse pup was subjected to SIMPLE (80 mg kg-1), and its spleen was dissected out, cut into pieces, and placed into a 24-well plate. The moving magnet led to a changing magnetic gradient.

Movie 12. The process of bleeding was recorded by two-photon cranial imaging. The distal middle cerebral artery of P6 mouse pup was occluded by SIMPLE for 6 hours. The white

rectangular box dictates the original location of hemorrhagic transformation and the leaking of blood cells from a vessel.

File name: Supplementary Data 1

Description: All the source data for directly generating statistical graphs and charts in Figure 1a', Figure 1h, Figure 3d, Figure 3h, Figure3I, Figure 3s, Figure 4k.
