# Supplementary material for: Precise control of embolic stroke with magnetized red blood cells in mice
Source: Commun Biol. 2022 Feb 16;5:136. doi: 10.1038/s42003-022-03082-9 (PMC8850623; doi:10.1038/s42003-022-03082-9)
Supplement: Supplementary file 6 — Reporting Summary [file 42003_2022_3082_MOESM6_ESM.pdf]

nature portfolio

Corresponding author(s): Jie-Min Jia

Last updated by author(s): Dec 15, 2021

Reporting Summary

Nature Portfolio wishes to improve the reproducibility of the work that we publish. This form provides structure for consistency and transparency in reporting. For further information on Nature Portfolio policies, see our Editorial Policies and the Editorial Policy Checklist.

Statistics

For all statistical analyses, confirm that the following items are present in the figure legend, table legend, main text, or Methods section.

n/a	Confirmed	
☐	☒	The exact sample size (*n*) for each experimental group/condition, given as a discrete number and unit of measurement
☐	☒	A statement on whether measurements were taken from distinct samples or whether the same sample was measured repeatedly
☐	☒	The statistical test(s) used AND whether they are one- or two-sided<br>*Only common tests should be described solely by name; describe more complex techniques in the Methods section.*
☒	☐	A description of all covariates tested
☒	☐	A description of any assumptions or corrections, such as tests of normality and adjustment for multiple comparisons
☐	☒	A full description of the statistical parameters including central tendency (e.g. means) or other basic estimates (e.g. regression coefficient) AND variation (e.g. standard deviation) or associated estimates of uncertainty (e.g. confidence intervals)
☐	☒	For null hypothesis testing, the test statistic (e.g. *F*, *t*, *r*) with confidence intervals, effect sizes, degrees of freedom and *P* value noted<br>*Give P values as exact values whenever suitable.*
☒	☐	For Bayesian analysis, information on the choice of priors and Markov chain Monte Carlo settings
☒	☐	For hierarchical and complex designs, identification of the appropriate level for tests and full reporting of outcomes
☐	☒	Estimates of effect sizes (e.g. Cohen's *d*, Pearson's *r*), indicating how they were calculated

Our web collection on statistics for biologists contains articles on many of the points above.

Software and code

Policy information about availability of computer code

Data collection	All TEM data were collected from TALOS L120C G2 or Tecnai T10 transmission electron microscopy; All SEM data were collected from Zeiss Gemini 550 or nova 450 scanning electron microscope. All wide-field images were collected from Zeiss Axio ZOOM.V16. The images of in vitro enrichment of magnetized red bleed cells were obtained from cell phone HUAWEI nova 2. All the immunofluorescence images were collected Zeiss LSM800 confocal microscope. All the MRI were obtained from BioSpec 70/20 USR, Germany. Laser speckle contrast imaging were collected from RWD RFLSI III.
Data analysis	All TEM and SEM images were processed by Velox and smartSE. All wide-field images and mmunofluorescence images were processed by Zeiss ZEN 2.3. All statistical analysis data resulted from GraphPad Prism 7.0. All the MRI images were exported by ParaVision 6.0.1. and analysis was done by using Image J. Images acquired by Laser speckle Contrast Imaging device were processed by LSCI_V1.0.0. All the rest of images were analyzed with Image J software.

For manuscripts utilizing custom algorithms or software that are central to the research but not yet described in published literature, software must be made available to editors and reviewers. We strongly encourage code deposition in a community repository (e.g. GitHub). See the Nature Portfolio guidelines for submitting code & software for further information.

Data

Policy information about availability of data

All manuscripts must include a data availability statement. This statement should provide the following information, where applicable:

- Accession codes, unique identifiers, or web links for publicly available datasets
- A description of any restrictions on data availability
- For clinical datasets or third party data, please ensure that the statement adheres to our policy

The source data underlying the graphs and charts shown in the figures and tables are provided in Supplementary Data 1. All data generated or analyzed during this study are included in this published article (and its supplementary information file).

Field-specific reporting

Please select the one below that is the best fit for your research. If you are not sure, read the appropriate sections before making your selection.

☒ Life sciences ☐ Behavioural & social sciences ☐ Ecological, evolutionary & environmental sciences

For a reference copy of the document with all sections, see nature.com/documents/nr-reporting-summary-flat.pdf

Life sciences study design

All studies must disclose on these points even when the disclosure is negative.

Sample size	For RBC magnetization efficiency measurements, n = 3 biological replicates were chosen as the minimal replicate number for the doses 0.2, 0.5, 1.5, and 2.0 pg/RBC. The minimal biological replicates were required to detect unpaired t-test significance with an alpha rate set as 0.05 in a standardly powered experiment. n = 1 for the RBC magnetization experiments of using the doses of 4 and 13.44 pg/RBC, which was performed alongside the lower dose mentioned above, because the RBCs were damaged so obviously compared to the lower dose that no further replicate needed. For cerebral blood flow measurements, the infarct size analysis, microglia/macrophage density determination, and GFAP, NeuN, and Iba1 imaging experiments, a minimum of 3 mouse pups were chosen. A minimal amount of mice were required to detect a p-value less than 0.05. req was determined once the statistical significance with an alpha rate set at 0.05. For probing the hemorrhagic transformation rate, more than the number of 10 mouse pups were chosen because it is the percentile data that belong to a binary variable (hemorrhagic or not hemorrhagic). Altogether, 250 mouse pups and 40 rat pups were used in this project. Those animals (11 mouse pups and 7 rat pups) with failed superficial injections were excluded in this study. No sample-size calculation was performed in this study.
Data exclusions	For RBC magnetization experiment, once the erythrocytes that lost a biconcave shape accounted for more than 10%, we ceased the process and initiated a separate mRBC preparation (Methods; paragraph 4). To perform SIMPLE and SIMPLeR models, animals that received failed superficial venous injections were excluded.
Replication	Each experiment presented in the study was repeated in multiple separate experiments or multiple animals (between 3- 41). All results in the paper are drawn from the analysis of multiple repeats and animals.
Randomization	Animals were assigned randomly to experimental and control groups.
Blinding	The investigators were not blinded to most of the experiments because the experimental readouts were very obvious, such as the effect of stroke on brain injury and the effect of magnetic field on magnetized RBC aggregation. However, the investigators were blind to the hemorrhagic transformation experiment when animals were subjected to the vehicle, minocycline, and PLX5622 treatments.

Reporting for specific materials, systems and methods

We require information from authors about some types of materials, experimental systems and methods used in many studies. Here, indicate whether each material, system or method listed is relevant to your study. If you are not sure if a list item applies to your research, read the appropriate section before selecting a response.

Materials & experimental systems

n/a	Involved in the study
☐	☒ Antibodies
☒	☐ Eukaryotic cell lines
☒	☐ Palaeontology and archaeology
☐	☒ Animals and other organisms
☒	☐ Human research participants
☒	☐ Clinical data
☒	☐ Dual use research of concern

Methods

n/a	Involved in the study
☒	☐ ChIP-seq
☒	☐ Flow cytometry
☐	☒ MRI-based neuroimaging

Antibodies

Antibodies used

Anti-Ter119-biotin, Cat# 13-5921-82, Lot# 2174196 and anti-GFAP, Cat# PA1-10004 were purchased from Thermo Fisher Scientific. Anti-NeuN, Cat# ABN90P, Lot# 3238431 was purchased from Millipore. Anti-Iba1, Cat# 019-19741 was purchased from Wako.

Validation

Anti-Ter119-biotin: Species from mouse; Applications for IHC, Flow, IF and ICC; Description: The TER-119 monoclonal antibody reacts with mouse erythroid cells from early proerythroblast to mature erythrocyte stages. The TER-119 antigen is present in yolk sac, fetal and newborn liver, but is not expressed by cells carrying BFU-E and CFU-E activities. Several erythroleukemia cell lines tested so far are negative for expression of TER-119 antigen even after dimethylsulfoxide stimulation. Biochemical and molecular analysis of the TER-119 antigen indicate that this molecule is associated with the surface glycophorin A, but is not a typical glycophorin; Reference: Yang Zhang., Development.2018 Feb 8;145(3):dev156745. doi: 10.1242/dev.156745.

Anti-GFAP: Species from Chicken; Applications for WB, IHC and IF; Description: GFAP (Glial fibrillary acidic protein) is a member of the class III intermediate filament protein family. GFAP is heavily and specifically expressed in astrocytes and certain astroglia of the central nervous system, in satellite cells of peripheral ganglia, and in non-myelinating Schwann cells of peripheral nerves. In addition, neural stem cells strongly express GFAP. Antibodies to GFAP are very useful as markers of astrocytic cells. In addition, many types of brain tumor, presumably derived from astrocytic cells, heavily express GFAP. GFAP is also found in the lens epithelium, Kupffer cells of the liver, in some cells in salivary tumors and has been reported in erythrocytes. GFAP is used as a marker to distinguish astrocytes from other glial cells during development. Mutations in this gene cause Alexander disease, a rare disorder of astrocytes in the central nervous system. Alternative splicing of the GFAP gene results in multiple transcript variants encoding distinct isoforms. Reference: Madlin Potratz., Cells.2020 Feb 11;9(2):412. doi: 10.3390/cells9020412.

Anti-NeuN: Species from guinea pig; Applications for WB, IHC , ICC and IF. Description: ABN90P is a Guinea Pig polyclonal version of the Anti-NeuN, clone A60 (MAB377), a highly characterized and cited mouse monoclonal antibody that specifically recognizes the DNA-binding, neuron-specific protein NeuN, which is present in most CNS and PNS neuronal cell types of all vertebrates tested. NeuN protein distributions are apparently restricted to neuronal nuclei, perikarya and some proximal neuronal processes in both fetal and adult brain although, some neurons fail to be recognized by NeuN at all ages: INL retinal cells, Cajal-Retzius cells, Purkinje cells, inferior olivary and dentate nucleus neurons, and sympathetic ganglion cells are some examples. Immunohistochemically detectable NeuN protein first appears at developmental timepoints that correspond with the withdrawal of the neuron from the cell cycle and/or with the initiation of terminal differentiation of the neuron. Immunoreactivity appears around E9.5 in the mouse neural tube and is extensive throughout the developing nervous system by E12.5. Strong nuclear staining suggests a nuclear regulatory protein function. No difference between protein isolated from purified nuclei and whole brain extract on immunoblots has been found. Reference: Jun Nishiyama., Neuron. 2017 Nov 15;96(4):755-768.e5. doi: 10.1016/j.neuron.2017.10.004. Epub 2017 Oct 19.

Anti-Iba1 Ionized calcium binding adaptor molecule 1 or Iba1 is specifically expressed in macrophages and microglia and upregulated during cell activation. Also known as allograft-Inflammatory Factor 1 (AIF1), Iba1 has actin-bundling activity and participates in membrane ruffling and phagocytosis in activated microglia. Wako's Anti-Iba1 polyclonal primary antibody was raised in rabbit using a synthetic peptide corresponding to the C terminus of Iba1 as immunogen. Anti-Iba1 is specific to microglia and macrophages but will not cross react with neurons or astrocytes.

Animals and other organisms

Policy information about studies involving animals; ARRIVE guidelines recommended for reporting animal research

Laboratory animals

Wide-type C57Bl6/J female and male healthy mice were used, the age ranged from 19 days at the embryo to 80 days after birth. Sprague Dawley female and male rats postnatal day 2-5 were used.

Wild animals

This study did not involve wild animals.

Field-collected samples

This study did not involve samples collected from the field.

Ethics oversight

All animal procedures complied with the guidelines of the Institutional Animal Care and Use Committee (IACUC) at the School of Life Sciences, Westlake University.

Note that full information on the approval of the study protocol must also be provided in the manuscript.

Magnetic resonance imaging

Experimental design

Design type	Our experiment did not involve tasks or testing state; event-related or block design. We used T2-weighted (T2-W) images the perinatal mice with stroke at different time points.
Design specifications	T2-W imaging of mouse head with coronal orientation was performed along the rostral to caudal axis.
Behavioral performance measures	The experimental process did not include behavioral performance.

Acquisition

Imaging type(s)	Structural imaging
Field strength	7.0 Tesla
Sequence & imaging parameters	Specify the pulse sequence type: spin echo; image type: RARE; Slices orientation: Axial; Read orientation: Le-Rt; Flip angle: 50°; Field of view: 15×15 mm2; Slice thickness: 0.5mm; Matrix size: 256×256.
Area of acquisition	Mouse whole brain

Diffusion MRI ☐ Used ☒ Not used

Preprocessing

Preprocessing software	All the MRI images were exported by the software of ParaVision 6.0.1.
Normalization	Not applied
Normalization template	Not applied
Noise and artifact removal	Not applied
Volume censoring	Not applied

Statistical modeling & inference

Model type and settings	Not applied
Effect(s) tested	Not applied

Specify type of analysis: ☒ Whole brain ☐ ROI-based ☐ Both

Statistic type for inference (See Eklund et al. 2016)	Not applied
Correction	Not applied

Models & analysis

n/a	Involved in the study
☒	☐ Functional and/or effective connectivity
☒	☐ Graph analysis
☒	☐ Multivariate modeling or predictive analysis
